# Mobile Genetic Element-Encoded Cytolysin Connects Virulence to Methicillin Resistance in MRSA

**DOI:** 10.1371/journal.ppat.1000533

**Published:** 2009-07-31

**Authors:** Shu Y. Queck, Burhan A. Khan, Rong Wang, Thanh-Huy L. Bach, Dorothee Kretschmer, Liang Chen, Barry N. Kreiswirth, Andreas Peschel, Frank R. DeLeo, Michael Otto

**Affiliations:** 1 Laboratory of Human Bacterial Pathogenesis, National Institute of Allergy and Infectious Diseases, The National Institutes of Health, Bethesda, Maryland, and Hamilton, Montana, United States of America; 2 Cellular and Molecular Microbiology Unit, Medical Microbiology and Hygiene Department, University of Tübingen, Tübingen, Germany; 3 Public Health Research Institute, University of Medicine and Dentistry of New Jersey, Newark, New Jersey, United States of America; Dartmouth Medical School, United States of America

## Abstract

Bacterial virulence and antibiotic resistance have a significant influence on disease severity and treatment options during bacterial infections. Frequently, the underlying genetic determinants are encoded on mobile genetic elements (MGEs). In the leading human pathogen *Staphylococcus aureus*, MGEs that contain antibiotic resistance genes commonly do not contain genes for virulence determinants. The phenol-soluble modulins (PSMs) are staphylococcal cytolytic toxins with a crucial role in immune evasion. While all known PSMs are core genome-encoded, we here describe a previously unidentified *psm* gene, *psm-mec*, within the staphylococcal methicillin resistance-encoding MGE SCC*mec*. PSM-mec was strongly expressed in many strains and showed the physico-chemical, pro-inflammatory, and cytolytic characteristics typical of PSMs. Notably, in an *S. aureus* strain with low production of core genome-encoded PSMs, expression of PSM-mec had a significant impact on immune evasion and disease. In addition to providing high-level resistance to methicillin, acquisition of SCC*mec* elements encoding PSM-mec by horizontal gene transfer may therefore contribute to staphylococcal virulence by substituting for the lack of expression of core genome-encoded PSMs. Thus, our study reveals a previously unknown role of methicillin resistance clusters in staphylococcal pathogenesis and shows that important virulence and antibiotic resistance determinants may be combined in staphylococcal MGEs.

## Author Summary

The extreme danger associated with *Staphylococcus aureus* infections is due to the combination of frequent antibiotic resistance, which prevents efficient treatment, with extraordinary virulence, which determines the severity of disease. *S. aureus* is known to exchange antibiotic resistance and virulence determinants between different strains, thereby spreading the capacity to cause serious infections in the *S. aureus* population. The genetic information for these determinants is usually found on so-called mobile genetic elements. It has been noted that such exchangeable elements carry genes for either virulence or antibiotic resistance, but not both. Here, we identified and characterized a potent toxin, whose gene is located within an element that encodes resistance to the important antibiotic methicillin. The toxin had strong capacity to kill human white and red blood cells and significantly affected the capacity of MRSA to cause disease. Our study shows that acquisition of methicillin resistance may be combined with gaining possession of potent toxins by a single event of genetic exchange, which likely represents an important feature accelerating the evolution of MRSA virulence.

## Introduction

Staphylococci are ubiquitous colonizers of human epithelia and frequent opportunistic pathogens involved in nosocomial infections [1]. In addition, the most virulent species, *Staphylococcus aureus*, can cause severe disease such as septicemia, toxic shock syndrome, and endocarditis, in both hospital and community settings [2].

The severity of a *S. aureus* infection is to a large extent determined by the toxin repertoire of the infecting strain. For example, *S. aureus* may produce toxic shock syndrome toxin-1 and other superantigens (enterotoxins), leukocidins, α-toxin, and phenol-soluble modulins (PSMs) [3],[4]. Many of these molecules destroy immune cells, thereby contributing considerably to the immune evasion capacity of *S. aureus*. Some toxins, such as α-toxin and the PSMs, are encoded on the bacterial core genome. Strain-to-strain differences in the secretion of these toxins are mainly due to differential gene expression [5]–[7]. In contrast, many other toxin genes are located on mobile genetic elements (MGEs). While many *S. aureus* strains produce MGE-encoded toxins, production of a given MGE-encoded toxin is usually limited to a small number of strains and strain-specific [8].

Staphylococcal infections are further complicated by frequent and sometimes multiple antibiotic resistance [9]. After the wide distribution of penicillinase-resistant strains in the middle of the last century [10], methicillin became the antibiotic of first choice for *S. aureus* infections. However, methicillin-resistant *S. aureus* (MRSA) are now prevalent in hospitals [11]. In addition, community-associated MRSA strains have emerged more recently and are spreading globally [12]. Furthermore, methicillin resistance is also common in coagulase-negative staphylococci such as *S. epidermidis* (methicillin-resistant *S. epidermidis*, MRSE) [13].

Antibiotic resistance genes are often located on MGEs such as transposons, plasmids, or genomic islands [14]. Specifically, the staphylococcal cassette chromosome *mec* (SCC*mec*) carries the *mecA* gene responsible for resistance to methicillin. There are at least 4 main and several sub-types of SCC*mec* elements, ranging from 21 to 67 kb in size, which are characterized by the two essential *mec* and *ccr* gene complexes and accessory gene loci such as transposons [14]. Importantly, while toxins and other virulence determinants are often encoded on MGEs, they have not been found within SCC*mec* elements or on widespread staphylococcal plasmids. Thus, acquisition of antibiotic resistance determinants by horizontal gene transfer in staphylococci is usually not linked to that of virulence factors [14].

PSMs are small, amphipathic and α-helical peptide toxins that attract and activate neutrophils [7]. In addition, PSMs of the α-type have pronounced capacity to lyse neutrophils and other cell types. Highly virulent CA-MRSA produce large amounts of the strongly cytolytic PSMα peptides, which are encoded in the core-genome located *psmα* operon and represent the main toxins contributing to neutrophil lysis in these strains. Deletion mutants in the *psmα* operon have dramatically reduced capacity to cause skin infections and bacteremia, indicating a crucial role of these toxins in *S. aureus* pathogenesis [7].

Here we identified and characterized an α-type PSM peptide that has pro-inflammatory and cytolytic activity and an important role in *S. aureus* infection. In contrast to all PSMs found so far, the newly identified *psm-mec* gene is encoded within an SCC*mec* MGE rather than on the core genome, providing a molecular connection between virulence and antibiotic resistance in staphylococci.

## Results

### Identification of the SCC*mec*-encoded PSM-mec

**Figure 1:**
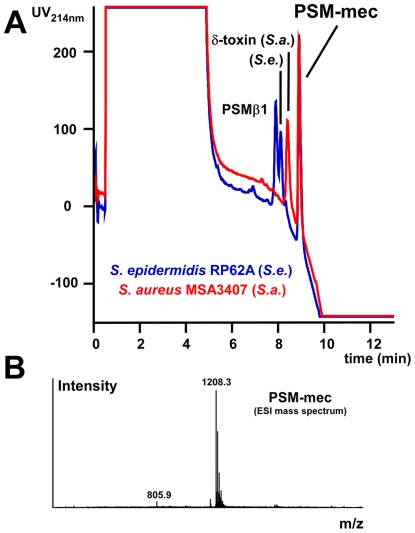
PSM-mec production in *S. aureus* and *S. epidermidis*. (A) RP-HPLC elution profile of *S. aureus* MSA3407 and *S. epidermidis* RP62A culture filtrates (at 8 h of growth). Equal volumes were applied to a C8 column and eluted with a 0.1% TFA water/acetonitrile gradient. PSM peptides characteristically elute at very high acetonitrile concentrations. Peaks of major PSM peptides and the newly identified PSM-mec are indicated. (B) Electrospray ion chromatogram of the PSM-mec peak obtained by RP/HPLC/ESI-MS (*S. epidermidis* RP62A). The respective PSM-mec ESI chromatogram of *S. aureus* MSA3407 or any strain with PSM-mec production showed the same m/z peaks. The series of peaks with slightly higher masses close to m/z 1208.3 are due to water and sodium adducts.

PSM peptides share physico-chemical properties rather than amino acid sequence similarity [7],[15],[16]. Additionally, *psm* genes are shorter than most cutoff thresholds for gene annotation. Therefore, to identify and classify a peptide as a member of the PSM family, initial detection and characterization of the peptide by means such as reversed-phase HPLC/mass spectrometry (RP-HPLC/MS), subsequent identification of the encoding gene, and detection of PSM-typical features such as amphipathy and α-helicity are required. Commonly, a given species produces a characteristic pattern of PSM peptides due to the fact that *psm* genes are encoded on the core genome rather than on MGEs [7],[15],[16]. However, while screening collections of *S. aureus* and *S. epidermidis* strains [17]–[21], we found that some strains showed an additional peptide peak in the RP-HPLC profile within the elution range characteristic for PSMs (shown for one *S. epidermidis* and one *S. aureus* strain in Figure 1A). The molecular weight of the peptide, 2414.6 Da (Figure 1B), as calculated from the electrospray ionization (ESI) mass spectrum obtained by RP-HPLC/ESI-MS, was the same in all these strains.

**Figure 2:**
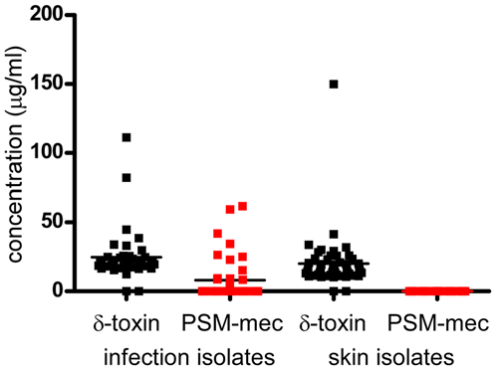
Production levels of PSM-mec in *S. epidermidis* infection and colonizing skin isolates. PSM-mec concentrations (at 8 h of growth) in *S. epidermidis* isolates from a Shanghai strain collection [19] obtained from the skin of healthy individuals without recent exposure to hospitals, or from *S. epidermidis* infections (mainly catheter-related, blood, and urinary tract infections) were determined by RP-HPLC/ESI-MS. Determined concentrations of δ-toxin in the strains are shown in comparison.

In detail, we analyzed a collection representing a wide variety of *S. aureus* strains [22], which contained 34 strains, 11 of which were MRSA, 79 MRSA strains of pulsed-field types USA100, USA200, USA300, USA500, USA1000, and USA1100 from infection and carriage isolates from San Francisco [17],[21], 54 infectious *S. epidermidis* strains from Paris, 56% of which were MRSE [23], and 180 *S. epidermidis* strains from Norway, 29% of which were MRSE [20]. Furthermore, we analyzed an *S. epidermidis* strain collection from Shanghai [19] that included 51 colonizing strains (no MRSE) and 41 isolates from infection (29% MRSE). 10% of all analyzed MRSA and 68% of all analyzed MRSE strains produced the peptide, while it was never detected in methicillin-sensitive *S. aureus* (MSSA) or *S. epidermidis* (MSSE). Accordingly, in the Shanghai collection, all producing *S. epidermidis* strains were isolated from human infections, whereas the MSSE skin isolates (colonizers) never produced the peptide (Figure 2). In the San Francisco strain collection, the peptide was found in 5 of 14 infectious USA100 and USA200 isolates, but never in other pulsed-field types. These results indicated that peptide production is linked to specific SCC*mec* elements.

**Figure 3:**
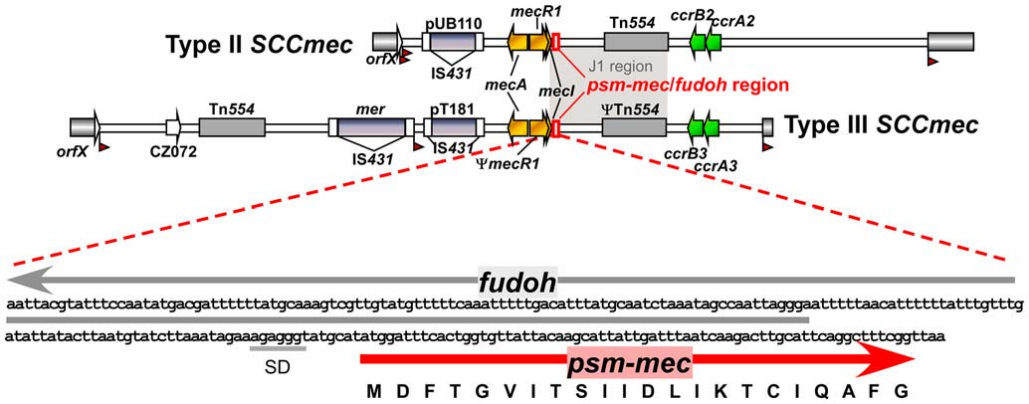
Location of the *psm-mec* gene in SCC*mec* elements of types II and III. Type II and III SCC*mec* elements are shown on the top, based on Genbank accession numbers D86934 (strain *S. aureus* N315, type II) and AB037671 (type III) [14]. The essential *mec* (methicillin resistance and regulation) and *ccr* (recombinase) genes are shown in color, other accessory elements are in grey. The region that contains the *psm-mec* gene and the overlapping *fudoh* locus [39] is magnified at the bottom. SD, Shine-Dalgarno sequence.

Next, we purified the peptide and determined the N-terminal sequence, which allowed identification of the peptide-encoding gene (Figure 3). Analysis of published staphylococcal genome sequences revealed presence of the gene in the type II SCC*mec* clusters of *S. epidermidis* strain RP62A [24], and *S. aureus* strains Mu50, N315 and Sanger 252 [25],[26] (Figure 3). In addition, a tblastn search (www.ncbi.nlm.nih.gov/blast.cgi) showed that the gene is present within SCC*mec* clusters of types II or III in a series of staphylococcal strains including strains of *S. aureus*, *S. epidermidis*, *S. saprophyticus*, *S. pseudintermedius*, and *S. sciuri*. Furthermore, we typed the analyzed MRSE producers from the Paris and Shanghai collections as predominantly of SCC*mec* type III (21/27) and the four MRSA producers from the Fitzgerald et al. collection [22] as SCC*mec* type II. Finally, we also detected the gene in MRSA strains from Canada and New York City (strains C10682 and BK20781, GenBank FJ390057 and FJ670542.1) that contain the novel SCC*mec* type VIII, which appears to have arisen from recombination between different SCC*mec* elements [27]. These results indicated that the gene is typically encoded in SCC*mec* elements, specifically in the J1 region that is common to SCC*mec* types II and III (Figure 3) [14]. Thus, we termed the novel PSM peptide PSM-mec owing to the fact that it is encoded within SCC*mec* clusters. Furthermore, presence of the gene in SCC*mec* types II and III is in accordance with the data obtained with different MRSA pulsed field types, particularly the absence from community-associated MRSA of pulsed-field type USA300, which contain SCC*mec* type IV [28].

**Figure 4:**
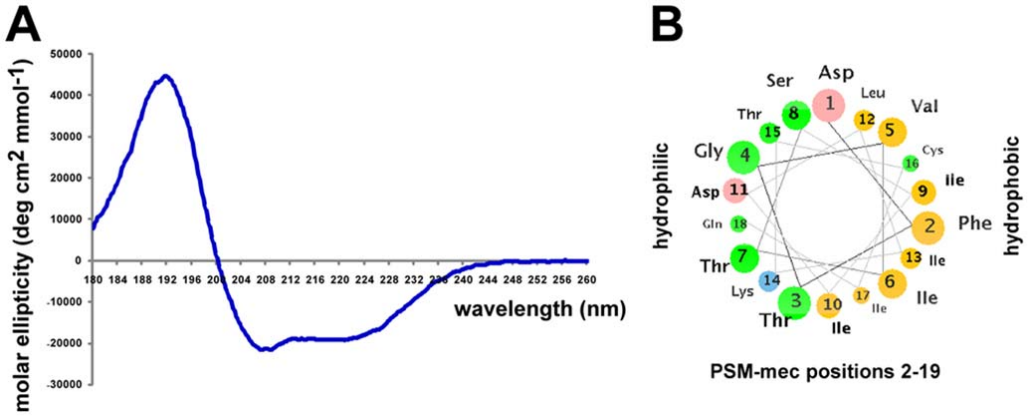
Physico-chemical characteristics of PSM-mec. (A) CD spectrum. Computation of α-helical content gave the following results: 59.3% (CONTINLL), 56.2% (SELCON3), 63.7% (CDSSTR). (B) Arrangement of PSM-mec (positions 2–19) in an α-helical wheel showing the amphipathy that is typical for PSMs: a hydrophilic side (left) and hydrophobic side (right).

Like all *psm* genes [7],[15],[16], the *psm-mec* gene contained only the DNA sequence encoding the final peptide product and no signal peptide. In addition, comparison of the theoretical mass of the translation product (2386.8 Da) with the detected mass of the secreted peptide indicated formylation of the N-terminal methionine (mass difference of 28 Da), which is common in bacterial proteins and found in all PSMs [7],[15],[16]. Analysis of secondary structure by circular dichroism (CD) (Figure 4A) and arrangement of the peptide sequence in an α-helical wheel (Figure 4B) revealed strong α-helicity and amphipathy, confirming that PSM-mec has characteristics typical of PSM peptides.

### Characteristics of PSM-mec production

**Figure 5:**
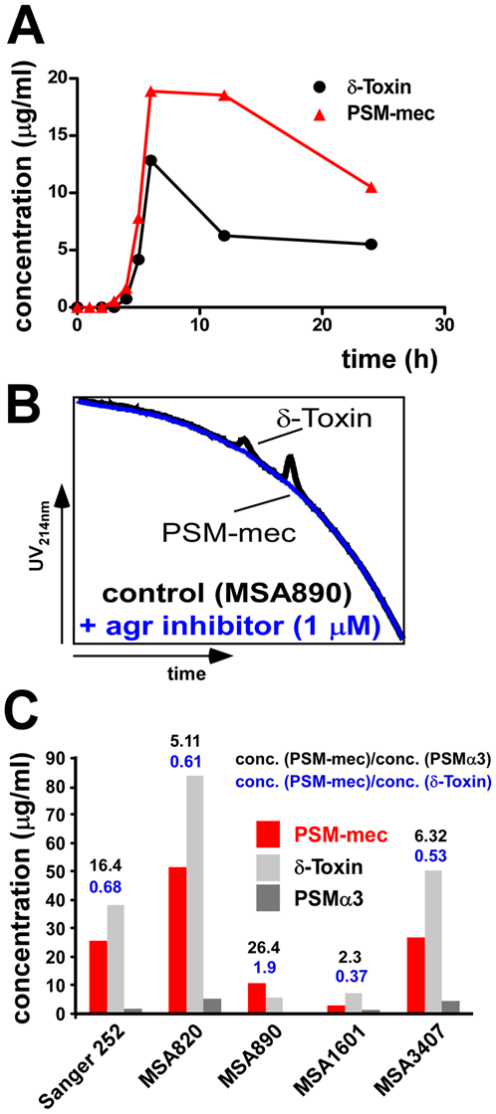
Characteristics of PSM-mec production and regulation in *S. aureus*. (A) Production of δ-toxin and PSM-mec during growth in shaken culture. (B) PSM production in strain *S. aureus* MSA890 with and without addition of *agr*-inhibiting *S. epidermidis* autoinducing peptide [30],[52]. With addition of inhibitor, no PSMs were detectable. (C) Production of selected PSMs at 8 h of growth in the PSM-mec producing *S. aureus* Sanger 252 and other MRSA strains [22].

All known PSM peptides are under control of the *agr* quorum-sensing system [7],[29]. Growth phase-dependent production of PSM-mec followed the same pattern as observed for other PSMs (Figure 5A), suggesting quorum-sensing control. Furthermore, the *agr*-dysfunctional MRSA strains N315 and Mu50 have all *psm* genes including *psm-mec* [26], but do not produce the corresponding gene products (data not shown). Moreover, we never detected PSM-mec in strains without δ-toxin production, which is indicative of a defective *agr* system. These observations suggested that PSM-mec production is dependent on *agr*. To further evaluate this hypothesis, we applied cross-inhibiting *S. epidermidis* autoinducing peptide, an efficient and specific inhibitor of *S. aureus agr* [30], to cultures of PSM-mec producing *S. aureus*. This led to complete absence of all PSMs, including PSM-mec (Figure 5B). Thus, PSM-mec production is under control by the quorum-sensing system *agr* like other PSMs.

The *psm* genes are regulated by direct binding of the AgrA response regulator to the *psm* promoter regions, distinguishing *psm* from many other *agr*-regulated genes that are under control of the regulatory RNA, RNAIII [31]. Analysis of the *psm-mec* promoter regions in the published genomes did not reveal consensus binding sites for AgrA (data not shown). However, as AgrA binding sites are not always completely conserved [31], the exact mechanism of *psm-mec* regulation by *agr* remains to be evaluated.

Notably, in many strains PSM-mec was produced at high levels, approximately achieving or in one strain exceeding production levels of the otherwise most abundant PSM, δ-toxin (PSMγ) (Figure 1, 2, 5C). Furthermore, while PSM-mec production was usually correlated with that of other PSMs, some strains showed a different production pattern. Strain MSA890 for example had high relative production of PSM-mec compared to other PSMs (Figure 5C). Thus, the fact that PSM-mec production is not always entirely correlated with that of δ-toxin indicates regulatory influences in addition to *agr*, as previously shown for other PSMs [7],[31].

### Role of PSM-mec in inflammation and immune evasion

**Figure 6:**
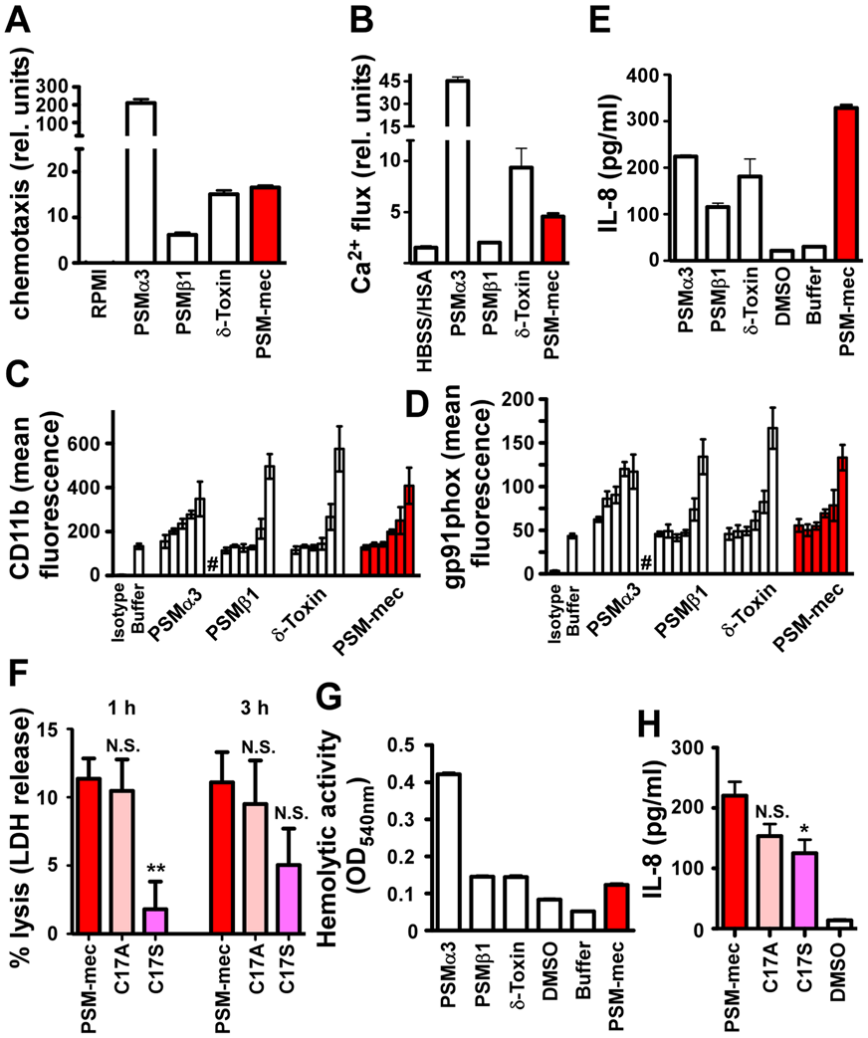
Pro-inflammatory and cytolytic capacities of PSM-mec and derivatives. (A) Chemotaxis of human neutrophils. Peptides were applied at 0.5 µg/ml (PSMα3), 2 µg/ml (δ-toxin), 5 µg/ml (PSM-mec), and 10 µg/ml (PSMβ1). (B) Calcium flux in human neutrophils. Peptides were applied at 0.1 µg/ml (PSMα3), 1 µg/ml (δ-toxin, PSM-mec), and 2.5 µg/ml (PSMβ1). In (A) and (B), values were corrected for the different concentrations applied. (C,D) Surface expression of CD11b and gp91*phox* on human neutrophils. #, lysis of neutrophils occurred. For PSMs, bars represent values obtained from increasing concentrations of peptide: 10, 100, 200, 400, 1000, 10000 ng/ml, from left to right in each group. (E) Secretion of the cytokine IL-8 at 10 µg/ml of PSM. (F) Neutrophil lysis at 50 µg/ml of PSM, comparison of PSM-mec with C17A and C17S replacement peptides. (G) Hemolytic activity. (H) Secretion of IL-8, comparison of PSM-mec with with C17A and C17S replacement peptides. (G,H) PSM peptides were applied at 10 µg/ml. (F,H) Statistical comparisons are vs. PSM-mec. *, p<0.05; **,p<0.01; N.S., not significant. In (A,B,C,D,E, and G) values for PSMs other than PSM-mec and controls that are shown for comparison are from Wang et al. [7]. Values for all samples including PSM-mec were obtained in parallel.

PSM peptides, particularly those of the α-type, cause chemotaxis, specific release of cytokines such as IL-8, and lysis of neutrophils and erythrocytes [7]. To analyze whether PSM-mec, which by its size and physico-chemical characteristics forms part of the PSM α-type family, has similar pro-inflammatory and lytic capacities, we first determined chemotaxis and calcium flux in human neutrophils. PSM-mec had lower chemotactic activities (Figure 6A) and elicited lower calcium flux (Figure 6B) than the most potent PSMα3, but in a range similar to that detected for other α-type PSMs and in general higher than that of β-type PSMs. Then, we determined the capacity of PSM-mec to activate human neutrophils by measuring surface exposure of gp91*phox* and CD11b (Figure 6C, 6D). Capacity of PSM-mec to activate human neutrophils was lower than that of the most potent PSMα3, but in the range of the other α-type PSMs and δ-toxin, and higher than that of β-type PSMs. The capacity of PSM-mec to elicit production of the cytokine IL-8 was somewhat higher than that detected for other α-type PSMs, but about in the same range (Figure 6E). Neutrophil lysis as likely the most crucial immune evasion property of PSMs was lower in PSM-mec than in other α-type PSMs. However, at 50 µg/ml, neutrophil lysis by PSM-mec obtained approximately the same level (Figure 6F) as observed previously for other α-type PSMs at 10 µg/ml [7]. Of note, these concentrations are typically achieved by many strains in vitro [7] (Figures 2, 5), indicating that the contribution of PSM-mec to overall cytolytic capacity of PSM-mec producing strains achieves that of PSMα peptides. Finally, lysis of sheep erythrocytes by PSM-mec was in an intermediate range compared to other PSMs (Figure 6G). Overall, these results demonstrate that PSM-mec has pro-inflammatory capacities similar to other α-type PSMs, although not as pronounced as for the most potent PSMα3.

### Role of the cysteine residue in PSM-mec

**Figure 7:**
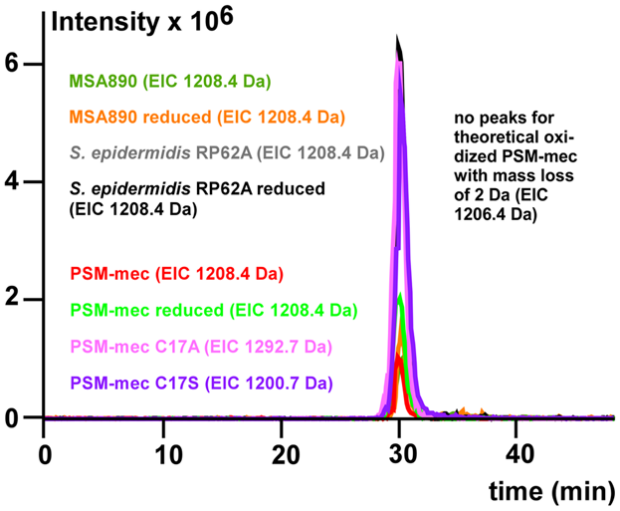
Analysis of PSM-mec dimerization by size exclusion chromatography/mass spectrometry (SEC/ESI-MS). Samples of synthetic PSMs and PSM-mec containing *S. aureus* or *S. epidermidis* culture filtrates, with or without reduction performed with 3% mercaptoethanol for 2 h at room temperature, were run on a Superdex HR 10/30 column with isocratic elution using 0.1% TFA/30% acetonitrile. Acid conditions in the elution buffer ascertain stability of the reduced or oxidized status during chromatography. The graphic shows the extracted ion chromatograms (EICs) of the main, double charged ions of the electrospray mass spectra of PSM-mec before and after reduction. No peaks were obtained when plotting EICs for a mass loss of 2 Da (1206.4 Da), as expected for dimeric, oxidized PSM-mec. In addition, extracted ion chromatograms of the alanine and serine subsitution peptides are shown (only one form owing to the absence of an oxidizable cysteine).

In contrast to all other PSM peptides identified until now, PSM-mec has one cysteine residue, which in secreted peptides usually indicates dimerization. To evaluate whether PSM-mec is present as a dimer and needs dimerization for its biological function, we synthesized mutant peptides with alanine and serine substitutions for the cysteine residue at position 17 of PSM-mec (PSM-mec C17A, PSM-mec C17S) (Figure 3). We first used size exclusion chromatography (SEC)/ESI-MS of PSM-mec C17A, PSM-mec C17S, and unreduced and reduced versions of PSM-mec to investigate whether PSM-mec is present as a dimer in its natural form. All peptides eluted at the same retention time (Figure 7). Furthermore, masses of the isolated and reduced versions of PSM-mec were the same and no peaks were obtained when calculating extracted ion chromatograms (EICs) with the mass of the theoretical oxidized, dimeric PSM-mec (Figure 7). Finally, EICs of *S. aureus* and *S. epidermidis* supernatants only showed the monomeric PSM-mec. These results indicate that PSM-mec does not dimerize despite the single cysteine in its amino acid sequence.

We then analyzed whether the cysteine residue is important for the biological function of PSM-mec, focusing on IL-8 secretion and neutrophil lysis. Substitution with alanine or serine only led to slightly reduced capacity to elicit production of IL-8 and lyse human neutrophils. Neutrophil lysis and IL-8 secretion were impaired to a more pronounced extent when the cysteine residue was replaced by serine than alanine, which is likely due to the fact that the cysteine residue is placed within the hydrophobic side of the amphipathic α-helix (Figure 4) and the hydroxyl side chain of serine may interfere with the amphipathic arrangement of the PSM-mec α-helix. These results indicated that the role of the cysteine residue in PSM-mec is likely limited to contributing to the PSM-mec α-helical structure and the cysteine sulfhydryl group does not have an additional specific function such as in peptide dimerization.

### PSM-mec has a limited impact on biofilm formation and intercellular aggregation

**Figure 8:**
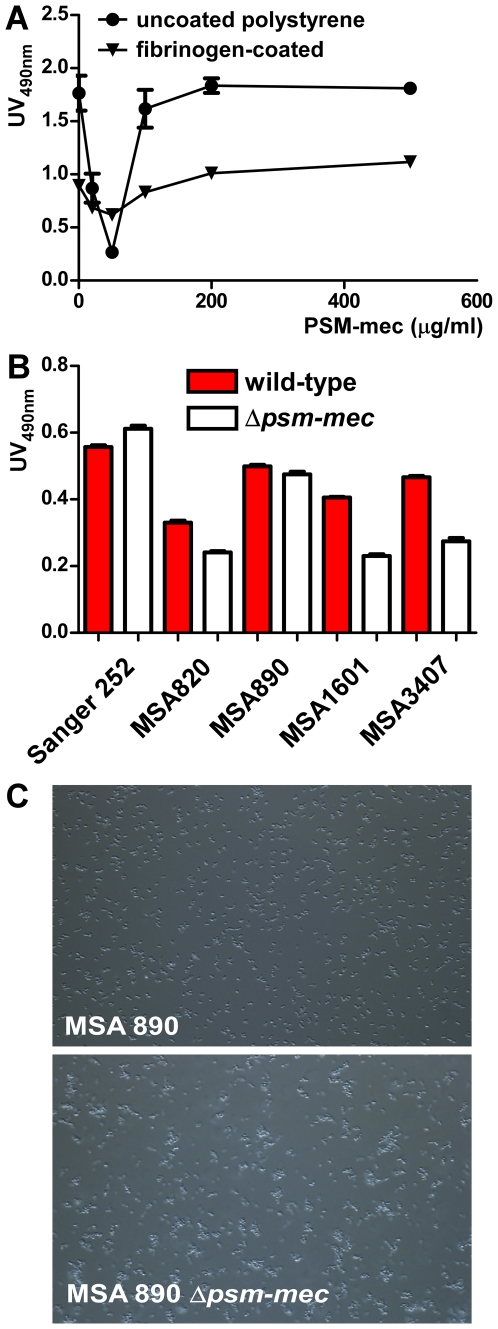
Influence of PSM-mec on biofilm formation and intercellular aggregation. (A) Impact of increasing concentrations of synthetic PSM-mec on biofilm formation using uncoated and fibrinogen-precoated polystyrene microtiter plates. PSM-negative *S. aureus* SA113 was used as indicator biofilm-forming strain. (B) Biofilm formation by *S. aureus* PSM-mec producers in comparison to isogenic *psm-mec* deletion mutants. (C) Aggregation phenotype of *S. aureus* MSA890 and isogenic *psm-mec* deletion mutant during mid-exponential growth phase (3 h) in shaken TSB flasks. Note that these are the same conditions as used to inoculate animals, in which owing to the aggregation phenotype, qRT-PCR and OD rather than CFU measurements were used to guarantee equal inocula. Differences in aggregation of the other isogenic strain pairs were similar or less pronounced.

PSM peptides have been suggested to impact biofilm formation based on their detergent-like structure that indicates surfactant capacities [32],[33]. To analyze whether PSM-mec influences biofilm development in *S. aureus*, we determined in vitro biofilm formation on microtiter plates. First, we added synthetic PSM-mec to the biofilm-positive, *agr*-negative strain SA113, which lacks PSM production (data not shown). We measured the impact of PSM-mec on biofilm formation directly on plastic and on fibrinogen-precoated plates, to mimic both possible mechanisms of attachment to indwelling medical devices [34] (Figure 8A). In both cases, there was reduced biofilm formation at intermediate PSM-mec concentrations (50 µg/ml), which corresponds to the range of PSM-mec production in bacterial culture filtrates. It is possible that the lack of biofilm-inhibiting activity at higher concentrations is due to peptide aggregation. Aggregation into micelle-like multi-molecular clusters in a concentration-dependent fashion has been described for the PSM δ-toxin [35].

To analyze the biological role of PSM-mec in vivo, we focused on *S. aureus* owing to its greater importance as a pathogen. We produced isogenic mutants by allelic replacement of the *psm-mec* gene in strains *S. aureus* Sanger 252 and the four MRSA strains from the analyzed *S. aureus* strain collection that showed PSM-mec production (MSA820, MSA890, MSA1601, MSA3407). Then, we compared the isogenic *psm-mec* deletion mutants with the corresponding wild-type strains. There were slight, yet significant influences on biofilm formation and intercellular aggregation in some strains (Figure 8B, 8C). Together, these results indicate that PSM-mec has a small concentration-dependent capacity to impact adhesion to surfaces, biofilm formation, and intercellular aggregation.

### PSM-mec contributes to pathogenesis

**Figure 9:**
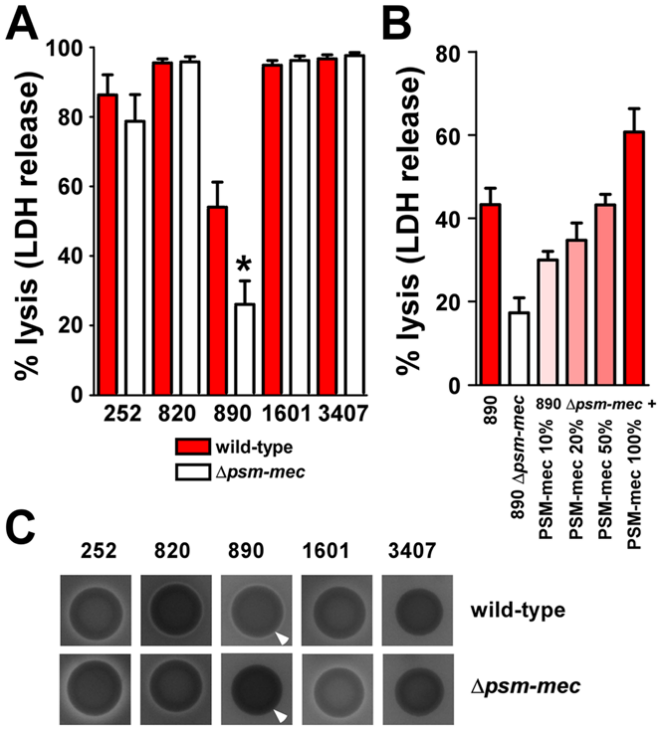
Cytolytic activities of *S. aureu*s PSM-mec producers and isogenic *psm-mec* deletion mutants. (A) Lysis of human neutrophils. Culture filtrates of strains were mixed with human neutrophils at a 1∶10 dilution and lysis was measured after 1 h by release of lactate dehydrogenase (LDH). Values are means±SEM obtained from neutrophils of 5 independent donors. *, p = 0.02 vs. wild-type. Differences between values for other wild-type and isogenic *psm-mec* deletion strains were not statistically significant. (B) Lysis of human neutrophils, complementation with PSM-mec. PSM-mec was added to culture filtrates of the *psm-mec* deletion strain in increasing concentrations (10, 20, 50, and 100% of the concentration detected in the wild-type strain). Experimental conditions were the same as in (A). Values are means±SEM obtained from neutrophils of 2 to 4 independent donors. (C) Synergistic hemolysis. Strains were grown for 24 h on sheep blood agar plates. Arrows mark different zones of synergistic hemolysis in strains *S. aureus* MSA890 and its isogenic *psm-mec* deletion mutant. (A,B) 252, *S. aureus* Sanger 252; 820, *S. aureus* MSA820; 890, *S. aureus* MSA890; 1601, *S. aureus* MSA1601; 3407, *S. aureus* MSA3407.

To investigate whether PSM-mec has a role in pathogenesis, we first analyzed neutrophil lysis caused by culture filtrates of the isogenic *psm-mec* deletion mutant strains compared to those of the corresponding wild-type strains. We detected significantly decreased capacity to lyse human neutrophils in the *psm-mec* deletion mutant of strain MSA890, but not in the other deletion strains (Figure 9A). Most likely, this is due to the fact that strain MSA890 produces considerably more relative amounts of PSM-mec, compared to core-genome encoded PSMs, than the other strains (Figure 5C). Addition of increasing concentrations of PSM-mec to culture filtrates of the MSA890 *psm-mec* deletion strain, up to 100% of that detected in the wild-type strain under corresponding growth conditions, completely restored the neutrophil-lytic capacity of the MSA890 wild-type strain (Figure 9B), ruling out the possibility that the observed phenotype was due to unintended second site mutations. Furthermore, pronounced synergistic hemolysis of strain MSA890, a phenotype caused by concerted activity of δ-toxin, other PSM or PSM-like peptides and α-toxin or β-toxin [36],[37], was considerably reduced by deleting the *psm-mec* gene in MSA890, whereas no marked reduction was detected in the other isogenic strain pairs (Figure 9C). These results indicate that PSM-mec production can substitute for the lack of cytolytic capacity in strains such as MSA890, in which expression of genome-encoded cytolytic PSMs is low. Notably, this includes lysis of human neutrophils as likely the most crucial function of PSMs in pathogenesis.

**Figure 10:**
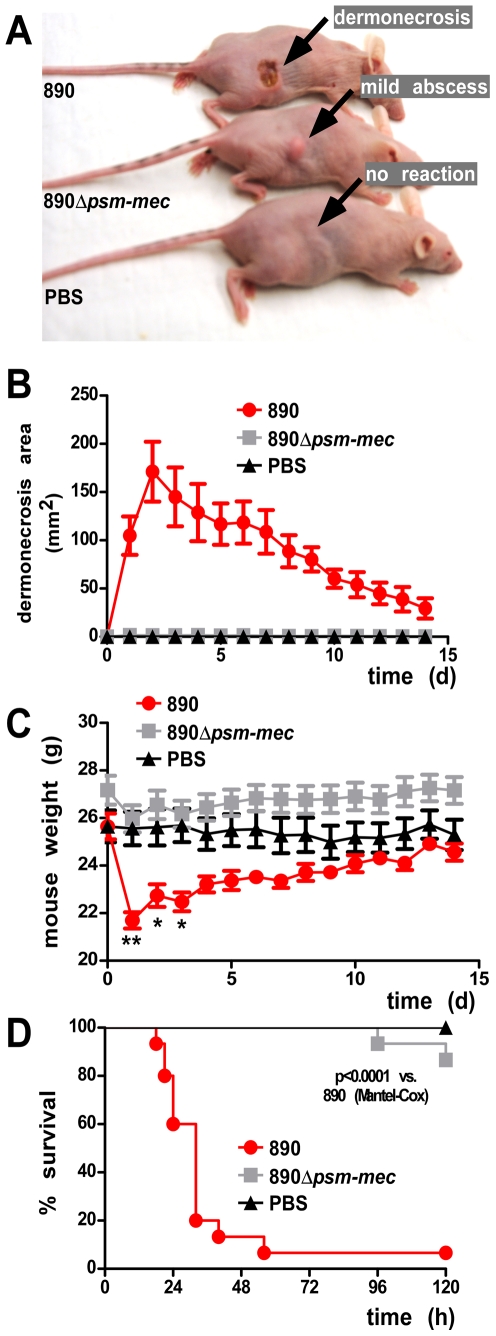
Impact of PSM-mec on virulence in animal infection models. (A–C) Mouse skin abscess model. Crl: SKH1-hrBR hairless mice were injected subcutaneously with 1×10^7^ CFUs/50 µl. Animal weights and skin lesion dimensions were examined at 24-h intervals for a total of 14 days. Number of mice: wild-type strain MSA890, 9; *psm-mec* deletion strain, 15, PBS control, 5. (A) Typical experimental outcome. Skin lesions developed in mice infected with the wild-type strain, while mice infected with the *psm-mec* deletion strain sometimes developed swelling/mild abscess formation at the infection site, but never lesions. Control mice injected with PBS showed no reaction. (B) Skin lesion sizes over time. (C) Animal weights over time. *, p<0.05; **, p<0.01, for mice infected with the wild-type strain compared to both other groups. (D) Mouse bacteremia model. CD1 Swiss female mice were injected with 1×10^8^ CFUs/100 µl and disease advancement was measured every 3 h for the first 24 h, then every 8 h for up to 72 h. Number of mice: wild-type strain MSA890, 15; *psm-mec* deletion strain, 15, PBS control, 5.

To analyze whether *psm-mec* impacts pathogenesis in important manifestations of *S. aureus* disease, we performed murine bacteremia and skin infection models. We have previously shown in these models that deletion of the strongly cytolytic α-type PSMs encoded on the *psmα* operon leads to greatly decreased potential of *S. aureus* to cause disease [7]. We selected the wild-type and *psm-mec* deletion mutant pairs of strains *S. aureus* MSA890 and Sanger 252, the latter as an example of the strains in which there was no change in cytolytic activity between *psm-mec* deletion and wild-type strains. With MSA890 and MSA890Δ*psm-mec*, we detected very significant differences in lesion size and weight loss in the skin infection model (Figure 10A–C) and in animal survival rates in the bacteremia model (Figure 10D). In contrast, there were no significant differences between strains *S. aureus* Sanger 252 and Sanger 252Δ*psm-mec* in the same models (data not shown). These results are in accordance with those achieved in the neutrophil lysis and hemolysis assays, indicating that the presence of PSM-mec may significantly impact *S. aureus* pathogenesis when PSM-mec levels exceed those of other cytolytic PSMs.

## Discussion

In this study, we identified a pro-inflammatory and cytolytic PSM peptide, PSM-mec, that is genetically linked to methicillin resistance, thus providing a molecular connection between two key traits determining the outcome of *S. aureus* disease. Our results indicate that acquisition of SCC*mec* elements encoding PSM-mec by horizontal gene transfer may significantly alter the capacity of *S. aureus* and potentially other staphylococci to cause disease, in addition to their established role in conferring resistance to methicillin. This represents a previously unknown example of toxin hitchhiking on staphylococcal mobile genetic elements primarily in charge of transferring antibiotic resistance.

While expression of the PSM-mec peptide did not significantly alter disease progression in a strain that produces high relative and absolute amounts of other cytolytic PSMs, PSM-mec had a very significant impact when other PSMs were only expressed at low levels, indicating that acquisition of *psm-mec*-encoding SCC*mec* elements may substitute for low level PSM expression in the recipient strain. Whereas PSM-mec expression is under control of *agr* as all other PSM peptides, additional regulatory factors are likely responsible for the high relative production of PSM-mec observed in strains such as *S. aureus* MSA890.

Using large strain collections, we did not detect PSM peptides other than PSM-mec and the previously described core genome-encoded PSMs. Therefore, PSM-mec is likely the only MGE-encoded staphylococcal PSM. The frequency of PSM-mec production was high in MRSE and considerable in MRSA of pulsed-field types USA100 and USA200, which represent the most common types associated with hospital infections [38].

Kaito et al. recently described an open reading frame called “*fudoh*” that is reportedly involved in colony spreading and virulence [39]. The 5′ end of *fudoh* overlaps to a large extent with the *psm-mec* gene, which is transcribed in opposite direction to *fudoh* (Figure 4). However, in contrast to *psm-mec*, there is no evidence for expression of *fudoh*. In addition, the *fudoh* gene does not have a Shine-Dalgarno sequence indicating a *fudoh* protein product is made. Possibly, the phenotypes attributed to *fudoh* by Kaito et al. [39] may thus have been due, at least in part, to interference with the *psm-mec* locus. Nevertheless, our results and those by Kaito et al. both emphasize the importance of the *fudoh*/*psm-mec* locus in *S. aureus* pathogenesis.

Finally, identification of PSM-mec may explain reports on a short-chain teichoic acid with pro-inflammatory properties described in *S. epidermidis* and termed “lipid S” [40]. The identification of “lipid S” as such was based on an electrospray mass spectrogram that showed exactly the same two m/z peaks as PSM-mec [40]. In contrast, it did not show the series of equidistant peaks commonly found for homopolymers such as teichoic acids. It is thus likely that the mass spectrograms leading to the description of “lipid S” were misinterpreted and the extracts contained PSM-mec. This would also explain a later report on pro-inflammatory capacities of “lipid S” [41].

In conclusion, our study shows that in contrast to previous belief, staphylococci may bundle resistance and virulence factors on mobile genetic elements, thus combining the transfer of two important determinants for causing human disease in one genetic exchange event.

## Materials and Methods

### Ethics statement

All animals protocols were reviewed and approved by the Animal Use Committee at Rocky Mountain Laboratories, NIAID, NIH. Human neutrophils were obtained from healthy volunteers in accordance with protocols approved by the Institutional Review Board for Human Subjects, NIAID, and the University of Tübingen, Germany.

### Strains and growth conditions

*S. aureus* and *S. epidermidis* genome sequencing strains (*S. epidermidis* RP62A and ATCC12228, *S. aureus* COL, Sanger 252, Sanger 476, N315, Mu50, USA300, and MW2) were acquired from the Network on Antimicrobial Resistance in *S. aureus* (NARSA). Other MRSE and MSSE *S. epidermidis* strains were from Shanghai (∼100 strains), Paris (∼70), and Norway (∼100) [18]–[20], and other *S. aureus* strains were from a San Francisco strain collection (∼80, all MRSA) in addition to those published by Fitzgerald et al. (∼35, MRSA and MSSA) [17],[21],[22]. All strains were grown in tryptic soy broth (TSB). When necessary during cloning of the allelic replacement plasmid, antibiotics were added at appropriate concentrations (ampicillin at 100 µg/ml for cloning in *E.coli*; chloramphenicol at 10 µg/ml for staphylococci). For strains for which information on methicillin resistance was not available from the literature, methicillin resistance was determined by plating on TSB agar containing 6 µg/ml oxacillin.

### Deletion of *psm-mec*

Allelic replacement of the *psm-mec* gene was performed using the procedure described by Bae and Schneewind [42] which allows for gene deletion without replacement by an antibiotic resistance cassette. Using this procedure, the *psm-mec* gene was deleted in its entirety. Briefly, 2 PCR fragments up- and downstream of *psm-mec*, introducing *att1* and *att2* recombination sites at the distal ends and an *Eco*RI site at the *psm-mec* ends were amplified from genomic DNA of *S. aureus* Sanger 252. Oligonucleotides used were PSMErev1 (caagacttgcattcaggctttcggtgaattctttc), PSMEatt1 (ggggacaagtttgtacaaaaaagcaggctggaagttttgtgctttataatgaacgggagcaagc), PSMErev2 (caccagtgaattccatatgcataccctctttc), and PSMEatt2 (ggggaccactttgtacaagaaagctgggtgtaccacctagcaaagttgcaaatttgac).

After digestion with *Eco*RI and ligation, the resulting fragment with flanking *att1* and *att2* sites was cloned into plasmid pKOR1 [42] using *att* recombination and a Clonase kit (Invitrogen). The resulting plasmid was electroporated in *S. aureus* RN4220, isolated from this strain and electroporated in the target strain. Afterwards, the allelic recombination procedure was performed as described [42]. Fidelity of gene deletion was determined by analytical PCR and RP-HPLC/ESI-MS. The PSM production phenotype of all deletion and wild-type strains was verified regularly and in all pre-cultures grown for key experiments using RP-HPLC/ESI-MS. This is important to rule out spontaneous mutation in the *agr* system, which happens frequently [43] and owing to *agr* control of all PSMs [7],[29],[31] may lead to strains completely devoid of PSM production.

### SCC*mec* typing

Typing of *S. epidermidis* and *S. aureus* SCC*mec* was performed using the method by Kondo et al. [44].

### Peptide synthesis

Peptides were synthesized by commercial vendors with an N-terminal formyl methionine residue in each peptide. Peptide sequence fidelity was determined by the Peptide Synthesis Unit of the NIAID.

### Circular dichroism (CD) measurement

The structures of synthetic PSM peptides were analyzed by CD spectroscopy on a Jasco spectropolarimeter model J-720 instrument. Solutions of PSM peptides, at 1.0 mg/ml, were prepared in 50% trifluoroethanol. Measurements were performed in triplicate and the resulting scans were averaged, smoothed, and the buffer signal was subtracted.

### Chromatography/mass spectrometry

RP-HPLC/ESI-MS was performed on an Agilent 1100 chromatography system coupled to a Trap SL mass spectrometer using a Zorbax SB-C8 2.3×30 mm column as described [29]. Quantification was performed by integration of the UV spectra, if peaks were well separated. Alternatively, quantification was based on extracted ion chromatograms using the most abundant peaks of the electrospray ion mass spectra of the respective PSM peptides, with calibration using synthetic peptides, as described [29]. SEC/ESI-MS was performed using the same equipment as RP-HPLC/ESI-MS with a Superdex Peptide HR 10/30 column (GE Healthcare) applying an isocratic gradient of 0.1% trifluoroacetic acid in 30% acetonitrile at 0.5 ml/min.

### PSM-mec purification and N-terminal sequencing

PSM-mec was purified from *S. epidermidis* RP62A stationary phase culture using the same procedure as used previously for the large-scale isolation of other PSMs [16]. Briefly, supernatant was precipitated using 10% ice-cold trichloroacetic acid. The pellet was dissolved in 100 mM Tris buffer pH 8.0 and taken to neutral pH with 6 N NaOH. Then, a 2-step reversed-phase chromatography protocol was used for purification as described [16]. For N-terminal sequencing at the Peptide Sequencing Unit of the NIAID, the N-terminal formyl group was removed by heating for 2 h at 55°C as described [45].

### Biofilm assays

Semi-quantitative biofilm assays using polystyrene microtiter plates and safranin staining were performed as described [46]. To assess the impact of PSM-mec on biofilm formation, the peptide was added at the time of inoculation with the indicator strain SA113 from pre-cultures (1∶100) at different concentrations. For pre-coating with fibrinogen, a 25 mg/l fibrinogen solution in phosphate-buffered saline (PBS) was filter-sterilized and 100 µl solution were pipetted in each well. After 18 h at 4°C, wells were washed twice with PBS, blocked with 2% sterile bovine serum albumin (BSA) solution for 2 h at 37°C, and washed 4 times with PBS. Then the biofilm assay was performed as described [46].

### Human neutrophil isolation

PMNs were isolated from venous blood of healthy volunteers as described [47],[48].

### Neutrophil chemotaxis and calcium ion fluxes

Neutrophils were subjected to a brief hypotonic shock with pyrogen-free water (Sigma), washed, and suspended at 5×10^6^ cells/ml in HBSS containing 0.05% human serum albumin (HSA) (CLB). Chemotaxis of neutrophils was determined by using fluorescently-labeled neutrophils that migrated through a membrane fitted into an insert of a 24-well microtiter plate transwell system (Costar) containing a prewetted 3-µm-pore-size polycarbonate filter as described [47]. For measurement of calcium ion fluxes, 5×10^6^ neutrophils/ml were loaded with 2 µM Fluo-3-AM (Molecular Probes) in RPMI containing 0.05% HSA (RPMI-HSA) for 20 min at room temperature under agitation, washed twice with buffer, and resuspended in RPMI-HSA at 10^6^ cells/ml. Calcium fluxes were analyzed with a FACScalibur (Becton Dickinson).

### Priming of human neutrophils

Priming of PMNs by synthetic PSMs was determined by increased surface expression of CD11b and gp91*phox* (granule exocytosis). PMNs were incubated with 10–10000 ng/ml PSMs in 96-well tissue culture plates at 37°C with rotation for 60 min. The assay was terminated by centrifuging cells at 4°C for 8 min at 350×g. Cells were washed twice in cold Dulbecco's phosphate-buffered saline and stained with and isotype control antibody (BD Biosciences) or those specific for CD11b (mAb 44, BD Biosciences) or gp91*phox* (mAb 7D5 [49]). Propidium iodide (0.5 µg/ml) was used to identify dead cells. PMNs were analyzed on a FACSCalibur flow cytometer (Becton Dickinson) and dead cells were excluded with a single gate. Percent positive neutrophils were determined with a marker defined by the boundary of the isotype-matched control antibody.

### Lysis of human neutrophils

Lysis of PMNs by synthetic PSMs or clarified *S. aureus* culture media was determined essentially as described [48],[50]. Synthetic PSMs (10 or 50 µg/ml) were added to wells of a 96-well tissue culture plate containing 10^6^ PMNs and plates were incubated at 37°C for up to 3 h. At the desired times, PMN lysis was determined by release of lactate dehydrogenase (LDH) (Cytotoxicity Detection Kit, Roche Applied Sciences). Alternatively, wild-type and isogenic mutant *S. aureus* strains were cultured for 24 h at 37°C in 50 ml TSB with shaking using a 100 ml flask. Bacteria were removed by centrifugation and culture media were sterilized by filtration and stored at −80°C in aliquots until used. Culture medium (diluted 1∶10) was mixed with human PMNs (10^6^) and tested for its ability to cause PMN lysis.

### Measurement of IL-8 production

Measurement of IL-8 production in human neutrophils was performed as described with a commercial ELISA assay kit (R&D systems) according to the manufacturer's instructions [7].

### Hemolysis

Hemolytic activity of PSM peptides was determined by incubating samples with a 2% (v/v) sheep red blood cells and incubation for 1 h at 37°C as described [7]. Hemolytic activity of *S. aureus* wild-type and *psm-mec* deletion strains was assessed by streaking on sheep blood agar plates.

### Mouse bacteremia and skin abscess models

Bacteremia and skin abscess models were performed as described [7]. Briefly, mice were between 4 and 6 weeks of age at the time of use. Mice were inoculated with *S. aureus* from mid-exponential growth phase (3 h) at ∼1×10^8^ CFUs/100 µl (bacteremia model) or ∼1×10^7^ CFUs/50 µl (abscess model) as described [48]. As strains showed different aggregation leading to different CFU, optical density was used to compare cell numbers and injection of equal cell numbers was verified by quantitative RT-PCR using the *gyrB* gene as described [51]. Control animals received sterile saline only.

For the bacteremia model, health and disease advancement of CD1 Swiss female mice were monitored every 3 h for the first 24 h, then every 8 h for up to 72 h. Animals were euthanized immediately if showing signs of respiratory distress, mobility loss, or inability to eat and drink. All surviving animals were euthanized at 72 hours.

For the abscess model, Crl: SKH1-hrBR hairless mice were examined for skin lesions and weight at 24-h intervals for a total of 14 days. Skin lesion dimensions were measured daily with a caliper. Length (*L*) and width (*W*) values were applied to calculate the area of lesions using the formula of *L* × *W*. All animals were euthanized after completion of the entire procedure.

### Statistics

Statistical analysis was performed using Student's t-tests for 2, or 1-way-ANOVA with Bonferroni post-tests for more than 2 groups, and Graph Pad Prism version 5 software.
